# Lifting the iron curtain of vision

**DOI:** 10.15252/emmm.202217259

**Published:** 2023-01-30

**Authors:** Boris Rosin, Jose‐Alain Sahel

**Affiliations:** ^1^ Department of Ophthalmology/UPMC Eye Center University of Pittsburgh Pittsburgh PA USA

**Keywords:** Metabolism, Signal Transduction

## Abstract

Ocular and specifically retinal toxicities of systemic medications are prevalent and encompass many disease modalities. For many of these pharmaceuticals, established follow‐up protocols are in place to ensure timely detection and cessation of therapy. However, while for some disorders, cessation of therapy is a viable option due to existing treatment alternatives, for some others cessation of treatment can be life threatening and/or shorten the patient's life expectancy. Such is the case for iron chelating agents used in transfusion‐dependent patients of Thalassemia, of which deferoxamine (DFO) is the most widely used. In their recent article in *EMBO Molecular Medicine*, Kong *et al* (2023) addressed the issue of DFO‐induced retinal toxicity used both *in vivo* and *in vitro* techniques. Their study suggests a potentially protective role for α‐ketoglutarate (AKG) supplementation against DFO toxicity.

## Graphical abstract

J.A. Sahel and B. Rosin discuss the study by Kong *et al*, in this issue of *EMBO Mol. Med.*, that shows that inhibiting HIF2α and protecting mitochondrial function via α‐ketoglutarate supplementation may effectively delay the progression of retinal degeneration in the clinic.

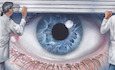

Retinal toxicities of systemic medications are a well‐established phenomenon. Two representative examples are hydroxychloroquine (HCQ)‐ and pentosan polysulfate (PSN)‐induced retinal toxicity. Notably, many more agents are known to be retinotoxic. For HCQ, an antimalarial used in rheumatological disease, the retinal side effects are well described, and monitoring guidelines are in place to ensure early detection (Marmor *et al*, 2016). The retinal toxicity of PSN, a drug commonly used in the treatment of interstitial cystitis, was described more recently (Pearce *et al*, 2018), and uniform screening guidelines are still being developed (Wang *et al*, 2020).

However, two important distinctions exist between the above agents and DFO‐related retinal toxicity. First, while alternative treatments exist for both HCQ and PSN, this is not the case for iron chelating agents in transfusion‐dependent Thalassemia patients. In these patients, iron chelation was shown to improve both the quality and the expectancy of life (Poggiali *et al*, 2012). Then, while both HCQ and PSN retinal toxicities are clinically relatively well described, this is not the case for their pathophysiology. To the best of our knowledge, PSN toxicity was not studied in animals to date. HCQ toxicity has been studied histologically in nonhuman primates (Rosenthal *et al*, 1978), but its pathophysiology, including the predilection of HCQ toward pigmented tissues, is very poorly understood (Sundelin & Terman, 2002).

In a first of a kind study of mechanisms of retinal toxicity, Kong *et al* examined the pathophysiological mechanisms underlying DFO toxicity in several models (Kong *et al*, 2023). First, they examined the effect of chronic DFO administration via intraperitoneal injections in wild‐type mice on both anatomical (optical coherence tomography, OCT as well as histological/immunohistochemical) and functional (electroretinography, ERG) levels and the effect was found to be dose dependent. As expected from clinical studies in patients, they concluded that the primary target tissue of DFO toxicity is the retinal pigment epithelium (RPE), evidenced by the differential effect on the c‐wave but not the a‐ and b‐waves of the mouse ERG mixed response.

Second, they proceeded to use both retinal preparations from DFO‐treated mice as well as RPE cells derived from human‐induced pluripotent stem cells (iPSCs), which they termed iRPE cells, to validate the results and further the understanding of the molecular mechanisms underlying DFO toxicity. Progressive dose‐dependent damage to the RPE was shown in retinal preparations. Morphological changes and loss of adhesion of iRPE cells in response to DFO were confirmed *in vitro*, albeit only at higher concentrations of DFO. The authors further showed that the nuclear concentration of one of the hypoxia inducible factors (HIF) isoforms, HIF2α, was enhanced in the DFO‐treated iRPE, whereas HIF1α was practically undetectable. The effect of HIF2α on iRPE cells was profound, as they upregulated the expression of genes that promoted cell death and glycolysis, disrupted iron homeostasis, and elevated reactive oxygen species (ROS) formation, with the latter already linked to HIF2α in other disorders (Singhal *et al*, 2021).

Finally, the authors examined the effects of AKG supplementation in DFO‐exposed animals and tissues. In DFO‐treated mice, the parallel use of AKG supplementation resulted in improved morphological (OCT) and functional (ERG) outcomes. While presenting a more complex picture with regard to ROS formation, it was shown that AKG does improve the preservation of mitochondrial capacity of iRPE cells. Finally, AKG supplementation suppressed the elevation of HIF2α levels following DFO treatment in mouse tissue preparations.

The importance of this work is two‐fold. First and foremost, it establishes the basis for potential prevention of DFO‐induced RPE and retinal toxicity by means of AKG supplementation. The latter, already used as a nutritional supplement (Gyanwali *et al*, 2022), is safe and widely available. Naturally, further safety studies are warranted for the use of AKG in the context described in this work. Interestingly, in one of the clinical examples in the foreword to their work, the authors note an 18‐month long history of AKG supplementation (used as a nutritional supplement), which coincided with subjective visual and objective functional improvement by ERG.

Second, and in more general terms, this work provides a new framework for addressing retinal toxicities, with the use of both iPSC‐derived target tissues and live animals or animal preparations to complement each other in furthering our understanding of the molecular pathophysiology of these toxicities and aiding in the search of viable prevention/treatment options. It is our hope that such approaches will provide insights into the mechanisms of retinal/RPE toxicity for many other pharmaceuticals, protecting the visual function of patients treated with such agents over the course of their treatment, while extending the clinical arsenal of their treating physicians. This is especially important for patients of disorders for which the retinotoxic pharmaceutical is the only viable treatment option (Fig 1).

**Figure 1:**
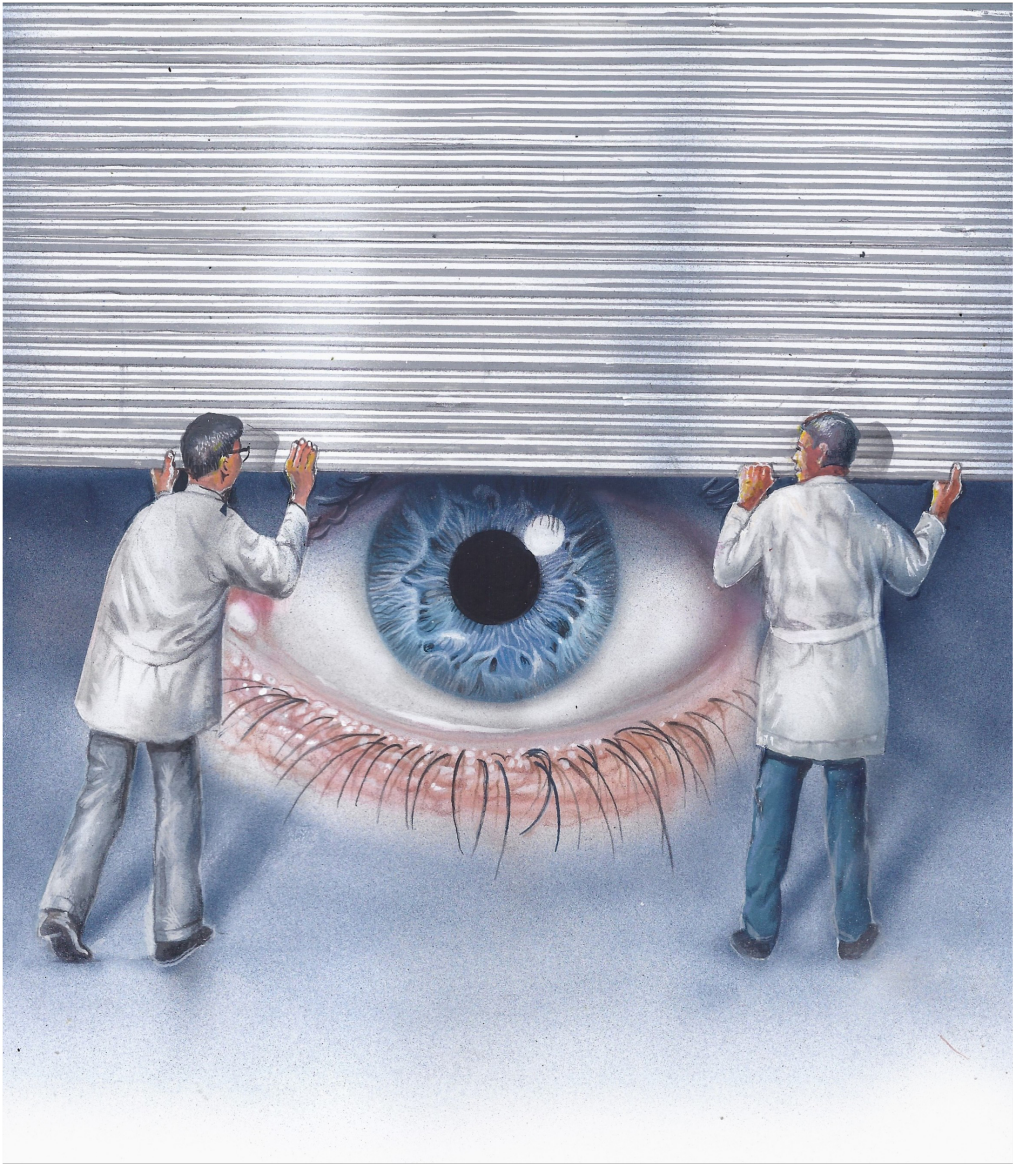
Fighting the retinal toxicity of DFO in transfusion‐dependent Thalassemia patients An illustration depicting the contribution of Kong *et al*, 2023. The retinal toxicity of DFO creates a “conflict of interests” between iron chelation in transfusion‐dependent Thalassemia patients, needed to improve the quality and the expectancy of their lives (Poggiali *et al*, 2012) and the retinal toxicity of DFO. This conflict could be viewed as a conceptual “iron curtain” over the vision of transfusion‐dependent Thalassemia patients. Kong *et al*, 2023 attempt to address this conflict through AKG supplementation, thus “lifting the iron curtain of vision”. Artist: Tuvia Kurtz.
